# Dendrobium officinale polysaccharides regulate age‐related lineage commitment between osteogenic and adipogenic differentiation

**DOI:** 10.1111/cpr.12624

**Published:** 2019-04-30

**Authors:** Hui Peng, Mi Yang, Qi Guo, Tian Su, Ye Xiao, Zhu-Ying Xia

**Affiliations:** ^1^ Department of Endocrinology, Endocrinology Research Center Xiangya Hospital of Central South University Changsha Hunan China

**Keywords:** BMSCs, Dendrobium officinale polysaccharides, osteoporosis, oxidative stress

## Abstract

**Objectives:**

Excessive oxidative stress and diminished antioxidant defences could contribute to age‐related tissue damage and various diseases including age‐related osteoporosis. Dendrobium officinale polysaccharides (DOPs), a major ingredient from a traditional Chinese medicine, have a great potential of antioxidative activity. In this study, we explore the role of DOP in age‐related osteoporosis that remains elusive.

**Materials and methods:**

Oxidative stimulation and DOP were used to treat bone marrow mesenchymal stem cells (BMSCs), whose lineage commitment was measured by adipogenic‐ and osteoblastic‐induced differentiation analysis. The oxidative stress and antioxidant capacity of BMSCs under the treatment of DOP were analysed by the level of MDA, SOD. Related mechanism studies were confirmed by qRT‐PCR, Western blotting and siRNA transfection. DOP was orally administrated in aged mice whose phenotype was confirmed by micro‐CT, immunofluorescence, immunochemistry and calcein double‐labelling analysis.

**Results:**

Dendrobium officinale polysaccharide treatment markedly increased osteogenic differentiation of BMSCs, while inhibiting adipogenic differentiation. In vitro, DOP could rescue H2O2‐induced switch of BMSCs differentiation fate. However, this effect was abolished in BMSCs when interfered with Nrf2 siRNA. Furthermore, administration of DOP to aged mice significantly increased the bone mass and reduced the marrow adipose tissue (MAT) accompanied with decreased oxidative stress of BMSCs.

**Conclusions:**

Our study reveals that DOP can attenuate bone loss and MAT accumulation through NRF2 antioxidant signalling, which may represent as potential therapeutic agent for age‐related osteoporosis.

## 1 INTRODUCTION

Osteoporosis is characterized by decreased bone mineral density and disrupted bone micro‐architecture. As a predominantly age‐related disease, it is becoming an emerging medical and socio‐economic threat as the progressive ageing population.^1^, ^2^ Accumulated evidences suggested that excessive oxidative stress and diminished antioxidant defences could contribute to age‐related tissue damage and various age‐related diseases, such as osteoporosis, osteoarthritis, diabetes, cardiovascular diseases and neurodegeneration.^3^, ^4^, ^5^, ^6^, ^7^, ^8^ It has been reported that oxidative stress is recognized as an independent factor involved in age‐related bone loss and could be associated with insufficient number of osteoblast and bone formation rate.^3^, ^9^, ^10^ Domazetovic et al demonstrated that oxidative stress impaired bone formation and subsequently contributed age‐related osteoporosis via attenuating osteoblastic differentiation.^11^ During ageing, bone marrow stem cells (BMSCs) tend to differentiate into adipocytes rather than osteoblasts, which subsequently lead to progressive MAT accumulation and bone loss.^10^, ^12^, ^13^ The imbalance between bone and fat in age‐related osteoporosis has been reported to be associated with an increasingly pro‐inflammatory tissue environment with mounting oxidative stress.^14^, ^15^, ^16^ Conversely, peroxisome proliferator–activated receptor coactivator 1‐a (PGC‐1a), with property of antioxidative defence, had an anti‐osteoporotic role though promoting osteogenesis and inhibiting adipogenesis.^14^

Dendrobium officinale, a traditional Chinese health medicine, has received much attention in recent years due to its pharmacological properties.^17^, ^18^ Dendrobium officinale polysaccharides (DOPs) are a major ingredient in Dendrobium officinale, which was reported to possess a wide range of potential effects including antioxidative, anti‐inflammatory, immunomodulation, anticancer, anti‐obesity, antihypertensive, hypoglycaemic and neuron protection activity.^19^, ^20^, ^21^, ^22^ Previous studies have shown that Dendrobium officinale extract can prevent the osteoporosis in ovariectomized mice.^23^ Moreover, ZHAO et al revealed that DOP has a great role in regulating cell fate of BMSCs.^24^ It could inhibit adipogenic differentiation in Rat BMSCs by deregulating adipogenesis‐related gene expression of PPARγ, LPL and FABP4.^24^ However, the role of DOP in age‐related bone loss and lineage commitment of BMSCs is still unclear.

Here, the present study demonstrates that DOP, as an antioxidative reagent, can attenuate oxidative stress damage and subsequently regulate age‐related BMSCs lineage commitment shift whose effects are related to Nrf2‐mediated antioxidative response. Taken together, our study provides potential therapeutic effect of DOP in age‐related osteoporosis.

## 2 MATERIALS AND METHODS

### 2.1 Mice

The 15‐month‐old mice were orally administrated with normal saline (NS) and DOP (150 mg/kg), respectively, once daily for 3 months. All protocols of animal care and experiment were reviewed and approved by the Institutional Animal Care and Use Committee of the Laboratory Animal Research Center at Xiangya Medical School of Central South University.

### 2.2 Mouse BMSCs isolate and cell culture

Mouse BMSCs isolation was described as before.^13^ Briefly, the cells were incubated with Sca‐1, CD29, CD45 and CD11b (BioLegend) after bone marrow cells were flushed from femora bone marrow cavity. Then, we sorted out Sca‐1 + CD29+CD45−CD11b– BM‐MSCs through flow cytometry. Next, cultured the cell with low‐glucose DMEM, 100  U/mL of penicillin/streptomycin and 10% FBS in a humidified atmosphere of 95% air and 5% CO_2_ at 37 °C.

### 2.3 Human bone marrow collection

We obtained the written informed consent from all participants before collecting bone marrow. The age of the participants was ranged from 20 to 79 years underwent hip replacement. All clinical bone marrows were approved by the Ethics Committee of the Xiangya Hospital of Central South University, and this study conformed to recognized standards.

### 2.4 Detection of cell viability

Cell viability was assessed by the methylthiazolyldiphenyl‐tetrazolium bromide (MTT) assay according to manufacturer's instructions. The absorbance was measured at 570 nm using a Multiskan™ GO microplate reader (Thermo Fisher Scientific).

### 2.5 Osteogenic differentiation and mineralization assay

BMSCs were cultured in 6‐well plates at a density of 2.5 × 10^6^ cells per well with osteogenesis induction medium (50 μg/mL ascorbic acid, 1 µmol/L dexamethasone and 5 mM β‐glycerophosphate) for 21 days.^25^

For ALP staining, we fixed the cell for 5 minutes with 10% formaldehyde, then incubated them for 2 hours at 37°C with ALP incubation buffer (0.2 g barbital sodium, 0.4 g magnesium sulphate, 0.2 g calcium chloride and 0.3 g beta‐glycerophosphate). Next, washed cells with 2% calcium chloride and incubated with 2% cobaltous nitrate for 5 minutes, then incubated them with 1:80 ammonium sulphate for 10 seconds. Finally, the cells were observed by microscope (Leica) and statistically calculated by Photoshop. We performed and quantified Alizarin red staining (Sigma‐Aldrich) to evaluate the cell matrix mineralization and observed by microscope (Leica) according to previous protocol.^13^

### 2.6 Adipogenic differentiation

BMSCs were cultured with adipogenesis induction medium (DMEM containing 10% FBS, 0.5 mmol/L 3‐isobutyl‐1‐methylxanthine, 5 μg/mL insulin and 1 μmol/L dexamethasone) for 14 days. We performed oil red O staining to detect mature adipocytes and quantified oil red released from the cell into the isopropanol solution by spectrophotometry at 540 nm according to previous protocol.^13^

### 2.7 Western blot and qRT‐PCR analysis

We performed Western blotting as previously described.^26^ The primary antibodies, NRF2 (ab62352; Abcam) or α‐tubulin (11224‐1‐AP; Proteintech), were incubated overnight, then incubated with appropriate HRP‐conjugated secondary antibodies for 1 hour at room temperature. The blots were visualized using ECL detection reagents. For qRT‐PCR analysis, total RNA from cultured cells was extracted using Trizol reagent (Invitrogen) and then performed qPCR using PrimeScript RT reagent Kit (Takara) and SYBR Green PCR Master Mix (Takara). Primer sequences are listed in Tables S1 and S2.

### 2.8 RNA interference experiments (siRNA)

Nrf2 siRNA was purchased from Riobio. Nrf2 siRNA was transfected into MCSs using Lipofectamine 2000 (Invitrogen) according to manufacturer's recommendations.

### 2.9 H_2_O_2_ treatment and antioxidant activity

Human MSCs were pre‐exposed to 100 μmol/L H_2_O_2_ for 2 hours. The level of SOD and MDA in BMSCs was determined by MDA assay kit and SOD assay kit (Nangjing Jiancheng Bioengineering Institution) following the instruction of manufacturer.

### 2.10 Micro‐CT analysis

The femurs were dissected from mice and fixed with 4% paraformaldehyde overnight, then scanned and analysed with high‐resolution micro‐CT (Skyscan 1172, Bruker MicroCT) as previously described.^13^ We selected the region of interest for analysis as 5% of femoral length below the growth plate. Trabecular bone volume per tissue volume (Tb. BV/TV), trabecular number (Tb. N), trabecular separation (Tb. Sp) and trabecular thickness (Tb. Th) were measured.

### 2.11 Immunohistochemistry analysis

Freshly, femora were dissected from mice, fixed overnight at 4℃ with 4% paraformaldehyde, decalcified in 10% EDTA (pH 7.4) for 21 days and then embedded in paraffin. Bone sections (4 μm thick, longitudinally oriented) were incubated with primary antibody against osteocalcin (Takara, M173) overnight at 4°C. Subsequently, an HRP‐streptavidin detection system (Dako) was used to detect the immunoactivity, followed by counterstaining with haematoxylin (Sigma‐Aldrich). Sections incubated with polyclonal rabbit IgG (R&D Systems) served as negative controls. Four randomly selected visual fields in the distal metaphysis of the femur were measured to test the number per millimetre of adjacent bone surface (Nmm^‐1^) in trabecular bone.

### 2.12 Calcein double labelling

We injected mice intraperitoneally with 0.08% calcein (Sigma‐Aldrich, 20 mg/kg bw) 8 and 2 days before euthanasia. Calcein double labelling in undecalcified bone slices was observed under a fluorescence microscope. Four randomly selected visual fields in the distal metaphysis of the femur were measured to test trabecular bone formation in femora.

### 2.13 Statistics

Data are presented as mean ± SD. We used two‐tailed Student's *t* test for 2‐group comparison and one‐way ANOVA for comparison within multiple groups. All experiments were conducted at least three times, and representative experiments were shown. *P* < 0.05 was considered as significant difference.

## 3 RESULTS

### 3.1 DOP stimulated osteoblast differentiation and inhibited adipocyte differentiation in BMSCs

DOP has been recognized as a valuable Chinese medicine due to its therapeutic effects. The cytotoxicity of DOP assessed by MTT assay indicated that it would not affect the cell viability at the concentrations of 100, 200 and 400 μg/mL (Figure S1A).

**Figure 1:**
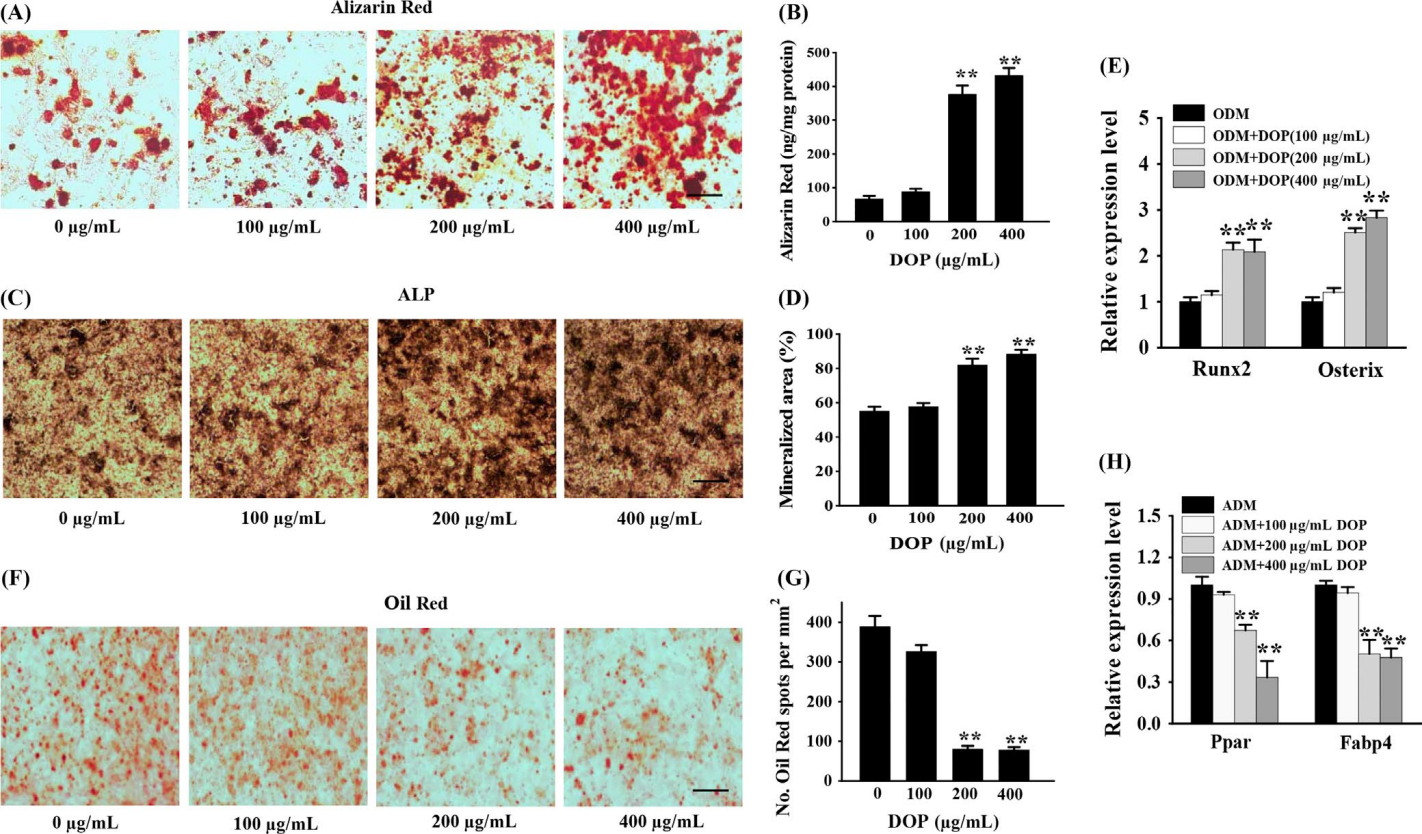
DOP stimulated osteoblast differentiation and inhibited adipocyte differentiation in BMSCs. (A‐D) BMSCs treated with osteogenesis differentiation medium (ODM) containing indicated concentrations of DOP for 21 d. Representative images of Alizarin red S staining (A) and quantitative analysis (B) of matrix mineralization in BMSCs. Scale bar: 100 μm. Representative images of ALP staining (C) and quantitative analysis (D) of matrix mineralization in BMSCs. Scale bar: 100 μm. (E) BMSCs treated with ODM containing indicated concentrations of DOP for 48 h. qRT‐PCR analysis of the relative levels of Runx2 and Osterix in BMSCs. (F and G) BMSCs treated with adipogenesis differentiation medium (ADM) containing indicated concentrations of DOP for 14 d. Representative images of oil red O staining of lipids (F) and quantification of the lipid droplet formation (G) in BMSCs. Scale bar: 100 μm. (H) BMSCs treated with ADM containing indicated concentrations of DOP for 48 h. qRT‐PCR analysis of the relative levels of Pparγ and Fabp4 in BMSCs. Data are represented as mean ± SD of three individual experiments (n = 3). Statistical significance was determined using analysis of variance (ANOVA). *P < 0.05. **P < 0.01

In order to investigate the effect of DOP in osteogenic differentiation of BMSCs, we treated the BMSCs with osteogenesis differentiation medium (ODM) supplement with DOP at different concentration, respectively. Then, the capacity of osteogenic differentiation of BMSCs was evaluated by ALP staining and Alizarin red staining. The results showed that the number of mineralized nodule and OD value in 200 and 400 μg/mL DOP groups were obviously increased compared to the 0 and 100 μg/mL DOP groups (Figure 1A‐D). Moreover, the qRT‐PCR analysis showed that the mRNA level of the osteoblast transcription factor Osterix and Runx2 was significantly elevated by DOP treatment in a dose‐dependent manner (Figure 1E). These results suggested that DOP facilitated osteogenic differentiation and mineral deposition of BMSCs. To investigate the effect of DOP in adipogenic differentiation, we cultured BMSCs with adipogenesis differentiation medium (ADM) supplement and treat them with DOP at different concentration, respectively. The results of oil red staining (Figure 1F) and quantification of lipid droplets (Figure 1G) showed DOP impaired adipogenic differentiation of BMSCs. Consistently, the qRT‐PCR analysis suggested that peroxisome proliferator–activated receptor‐g (Pparγ) and fatty acid–binding protein 4 (Fabp4), two key markers of adipocyte differentiation, were both significantly inhibited by DOP in a dose‐dependent manner (Figure 1H). Together, these results suggested that DOP stimulated osteoblastic differentiation and suppressed adipocytic differentiation of BMSCs.

### 3.2 DOP regulated differentiation of BMSCs through activating Nrf2 signalling

**Figure 2:**
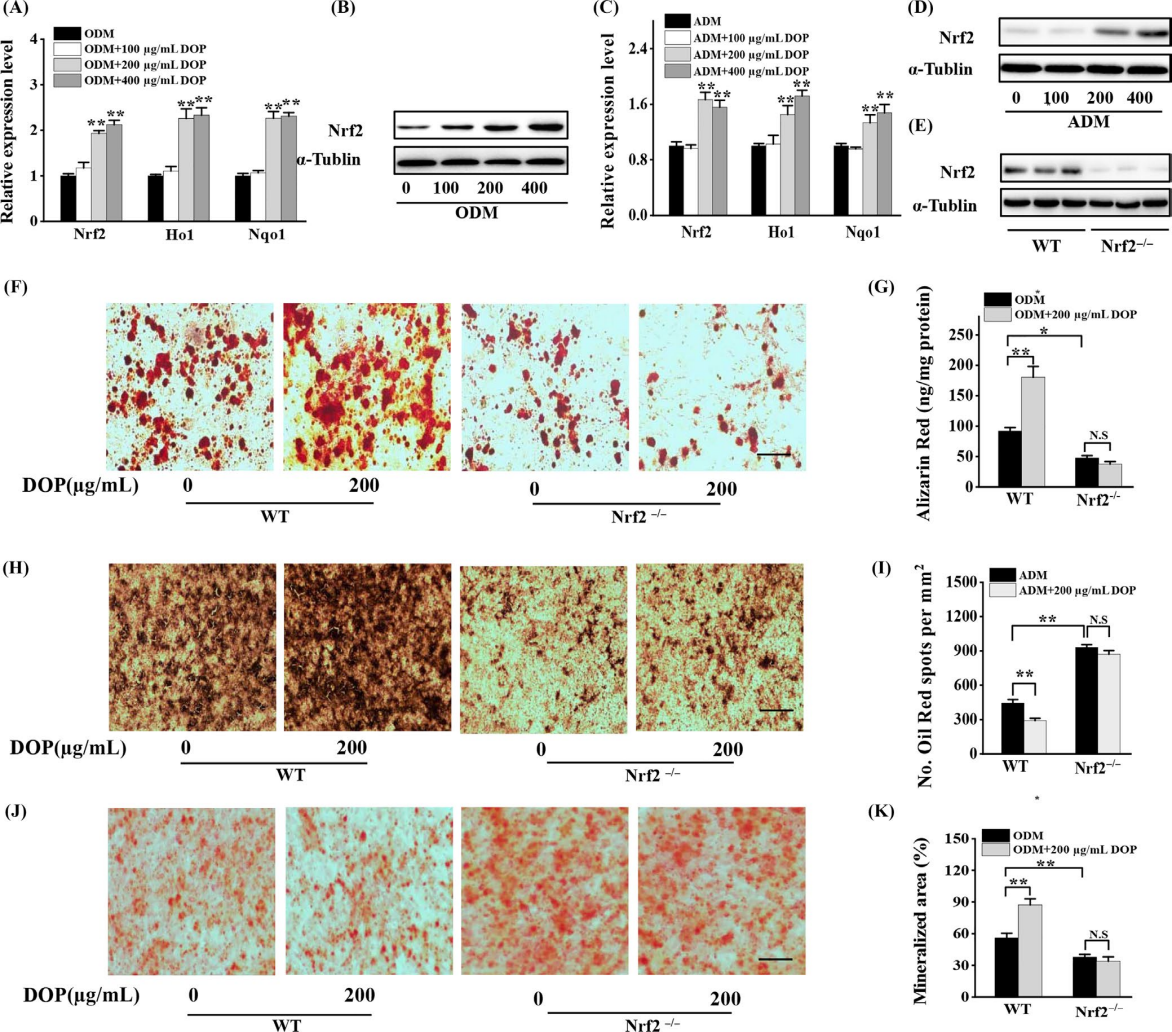
DOP regulated differentiation of BMSCs through activating Nrf2 signalling. (A and B) BMSCs treated with ODM containing indicated concentrations of DOP for 48 h. qRT‐PCR analysis of the relative levels of NRF2 and antioxidative enzymes (A) and Western blot analysis of the relative levels of NRF2 (B). α‐Tubulin was used as loading control. (C and D) BMSCs treated with ADM containing indicated concentrations of DOP for 48 h. qRT‐PCR analysis of the relative levels of NRF2 and antioxidative enzymes (C) and Western blot analysis of the relative levels of NRF2 (D). α‐Tubulin was used as loading control. (E) Western blot analysis of the relative levels of NRF2 in BMSCs transfected with siRNA targeting NRF2. (F‐I) BMSCs treated with ODM containing indicated concentrations of DOP for 21 d. Representative images of Alizarin red S staining (F) and quantitative analysis (G) of matrix mineralization in BMSCs. Scale bar: 100 μm. Representative images of ALP staining (H) and quantitative analysis (I) of matrix mineralization in BMSCs. (J and K) BMSCs treated with ADM containing indicated concentrations of DOP for 14 d. Representative images of oil red O staining of lipids (J) and quantification of the lipid droplet formation (K) in BMSCs. Scale bar: 100 μm. Data are represented as mean ± SD of three individual experiments (n = 3). Statistical significance was determined using analysis of variance (ANOVA) for A and C, and Student's *t* test for G, I and K. *P < 0.05, **P < 0.01. N.S, no significance

**Figure 3:**
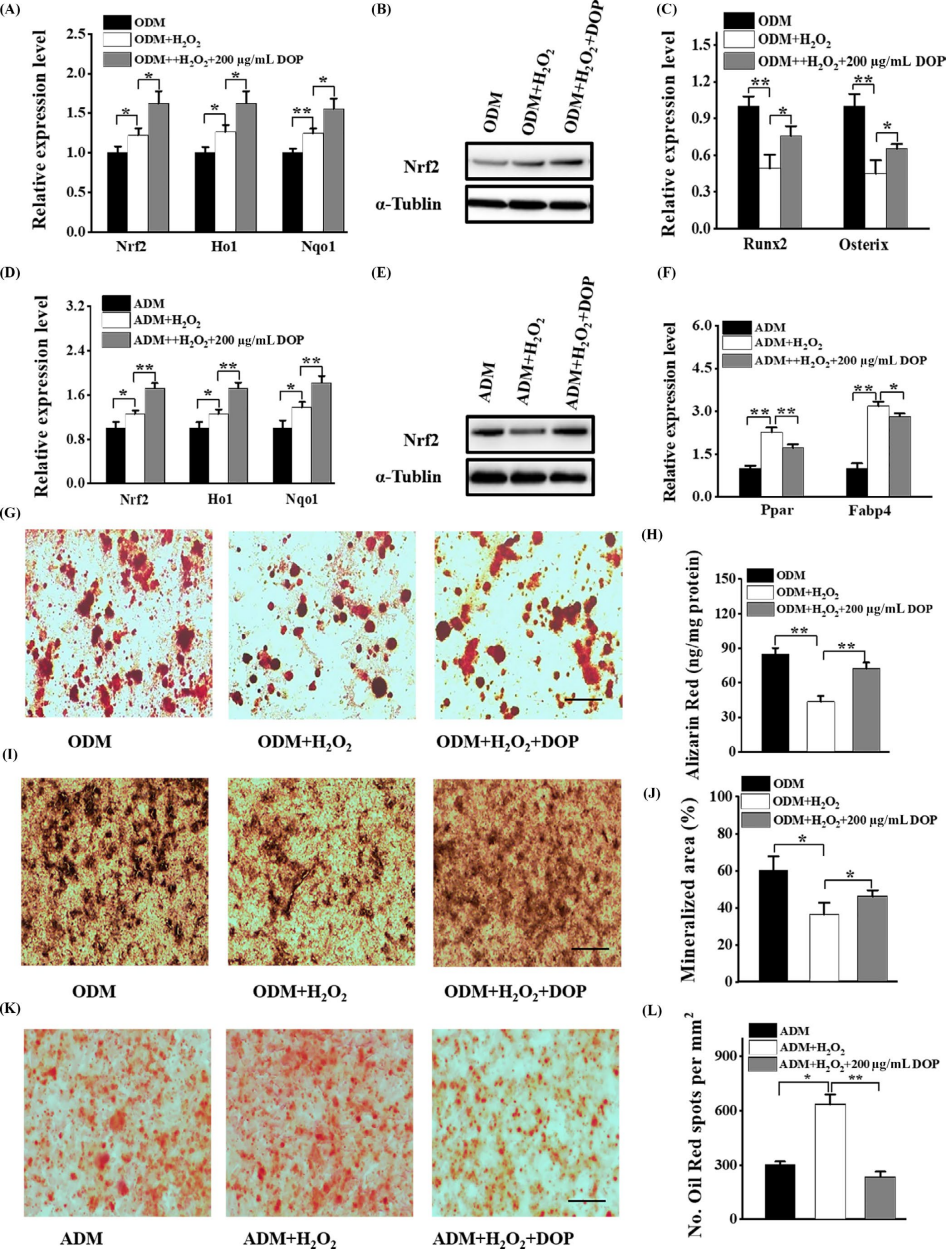
DOP restored oxidative stress–induced damage during osteoblasts and adipocyte differentiation. (A‐C) BMSCs were pre‐treated with 100 μmol/L H_2_O_2 _for 2 d and incubated in ODM with 200ug/mL DOP. (A) qRT‐PCR analysis of the relative levels of NRF2 and antioxidative enzymes in BMSCs. (B) Western blot analysis of the relative levels of NRF2 in BMSCs. (C) qRT‐PCR analysis of the relative levels of osteogenic‐related gene. (D‐F) BMSCs were pre‐treated with 100 μmol/L H_2_O_2_ for 2 d and incubated in ADM with 200 µg/mL DOP. (D) qRT‐PCR analysis of the relative levels of NRF2 and antioxidative enzymes in BMSCs. (E) Western blot analysis of the relative levels of NRF2 in BMSCs. (F) qRT‐PCR analysis of the relative levels of adipogenic‐related gene. (G‐J) BMSCs were pre‐treated with 100 μmol/L H_2_O_2_ for 2 d and incubated in ODM with 200 µg/mL DOP for 21 d. Representative images of Alizarin red S staining (G) and quantitative analysis (H) of matrix mineralization in BMSCs. Scale bar: 100 μm. Representative images of ALP staining (I) and quantitative analysis (J) of matrix mineralization in BMSCs. Scale bar: 100 μm. (K and L) BMSCs were pre‐treated with 100 μmol/L H_2_O_2_ for 2 d and incubated in ADM with 200 µg/mL DOP for 14 d. Representative images of oil red O staining of lipids (K) and quantification of the lipid droplet formation (L) in BMSCs. Scale bar: 100 μm. Data are represented as mean ± SD of three individual experiments (n = 3). Statistical significance was determined using analysis of variance (ANOVA). *P < 0.05. **P < 0.01

DOP has been reported to be associated with antioxidative activity through NRF2 signalling pathway.^27^ NRF2, as a master regulator of the cellular antioxidant response, is involved in regulating lineage switch between osteogenic and adipogenic fate of BMSCs.^28^ qRT‐PCR result showed a markedly elevated expression of Nrf2 mRNA in BMSCs cultured in ODM treated with 200 µg/mL or 400 µg/mL DOP (Figure 2A). The expression level of Ho1 and Nqo1, as antioxidative enzymes of Nrf2 downstream, also significantly increased under DOP treatment (Figure 2A). Western blot analysis revealed that DOP had a great role in elevating Nrf2 expression (Figure 2B). Consistently, BMSCs cultured in ADM and under the treatment of DOP also showed the same antioxidative tendency in mRNA and protein level of Nrf2 (Figure 2C,D). These results showed that DOP increased the expression of Nrf2 in BMSCs during both osteogenesis and adipogenesis. To validate whether DOP modulates cell fate of BMSCs through the activation of Nrf2, we treated the BMSCs with DOP and silenced Nrf2 by siRNA. The Western blot result confirmed the successful interference of Nrf2 (Figure 2E). Silencing of NRF2 inhibited osteogenic differentiation of BMSCs measured by Alizarin red staining (Figure 3F,G) and ALP staining (Figure 2H,I), while stimulating adipogenic differentiation of BMSCs measured by oil red staining (Figure 2J,K). DOP treatment could increase osteogenic differentiation and inhibit adipogenic differentiation of BMSCs in WT group. However, the protective effects of DOP could not rescue the imbalance between osteoblasts and adipocytes differentiation in Nrf2‐knock‐down BMSCs (Figure 2F‐K). These data supported the hypothesis that DOP attenuated the adipogenic differentiation and promoted osteogenic differentiation in BMSCs through Nrf2 signalling.

### 3.3 DOP restored oxidative stress–induced damage during osteoblastic and adipogenetic differentiation

**Figure 4:**
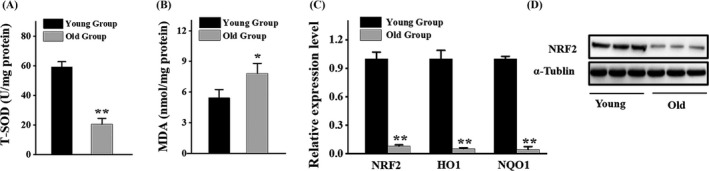
NRF2 expression level in human BMSCs was decreased during ageing. BMSCs were isolated from human male bone marrow at different ages. All samples were classified into two groups according to patients' age (n = 5 per group): young group (age: <30); old group (age > 50). (A) Superoxide dismutase (SOD) level in human BMSCs (hBMSCs). (B) Malondialdehyde (MDA) level in hBMSCs. (C) qRT‐PCR analysis of the levels of NRF2, HO1, NQO expression in hBMSCs. (D) Western blot analysis of the levels of NRF2 expression in hBMSCs. Data are represented as mean ± SD of three individual experiments (n = 5). Statistical significance was determined using Student's *t* test. *P < 0.05. **P < 0.01

To further confirm the antioxidative activity of DOP, we treated BMSCs with or without H_2_O_2 _at the concentration of 200 µmol/L for 2 hours to induce oxidative stress, and then the culture medium was supplemented with indicated concentration of DOP. The mRNA level and protein level of Nrf2 and antioxidative enzymes in BMSCs were obviously increased by the treatment of DOP under oxidative stress (Figure 3A,B). The osteogenic markers were significantly decreased in H_2_O_2_ pre‐treated BMSCs during osteogenic differentiation. However, this phenomenon was significantly rescued by DOP treatment (Figure 3C). Consistently, the qRT‐PCR and Western blot analysis revealed that DOP could increase the expression of Nrf2 and antioxidative enzymes in BMSCs when cultured in ADM (Figure 3D,E). Additionally, the qRT‐PCR analysis suggested that the expression of Pparγ and Fabp4 was increased under H_2_O_2_‐induced oxidative stress, which could be restored by DOP (Figure 3F). The important role of DOP in modulating the oxidative stress–impaired osteogenesis and oxidative stress–enhanced adipogenesis in BMSCs was further confirmed by Alizarin red staining (Figure 4G,H), ALP staining (Figure 4I,J) and oil red staining (Figure 4K,L).

### 3.4 NRF2 expression level in human BMSCs was decreased during ageing

Ageing is always accompanied with increased oxidative stress. We isolated and cultured human BMSCs (hBMSCs) from male subjects at different ages. Compared to young group, the old group represented a remarkable decrease in SOD activity which indicated antioxidative capability (Figure 4A) and an increase in MDA level which indicated oxidative stress level (Figure 4B). These data implied an elevated level of oxidative stress in hBMSCs during ageing. Specifically, the expression of NRF2 was notably downregulated with ageing both at mRNA level and protein level (Figure 4C,D). Alternatively, qRT‐PCR analysis revealed that antioxidative enzymes were correlated with NRF2 (Figure 4C). These results support that antioxidant NRF2 signalling plays an important role in the ageing process.

### 3.5 DOP treatment attenuated bone loss and MAT accumulation in aged mice

**Figure 5:**
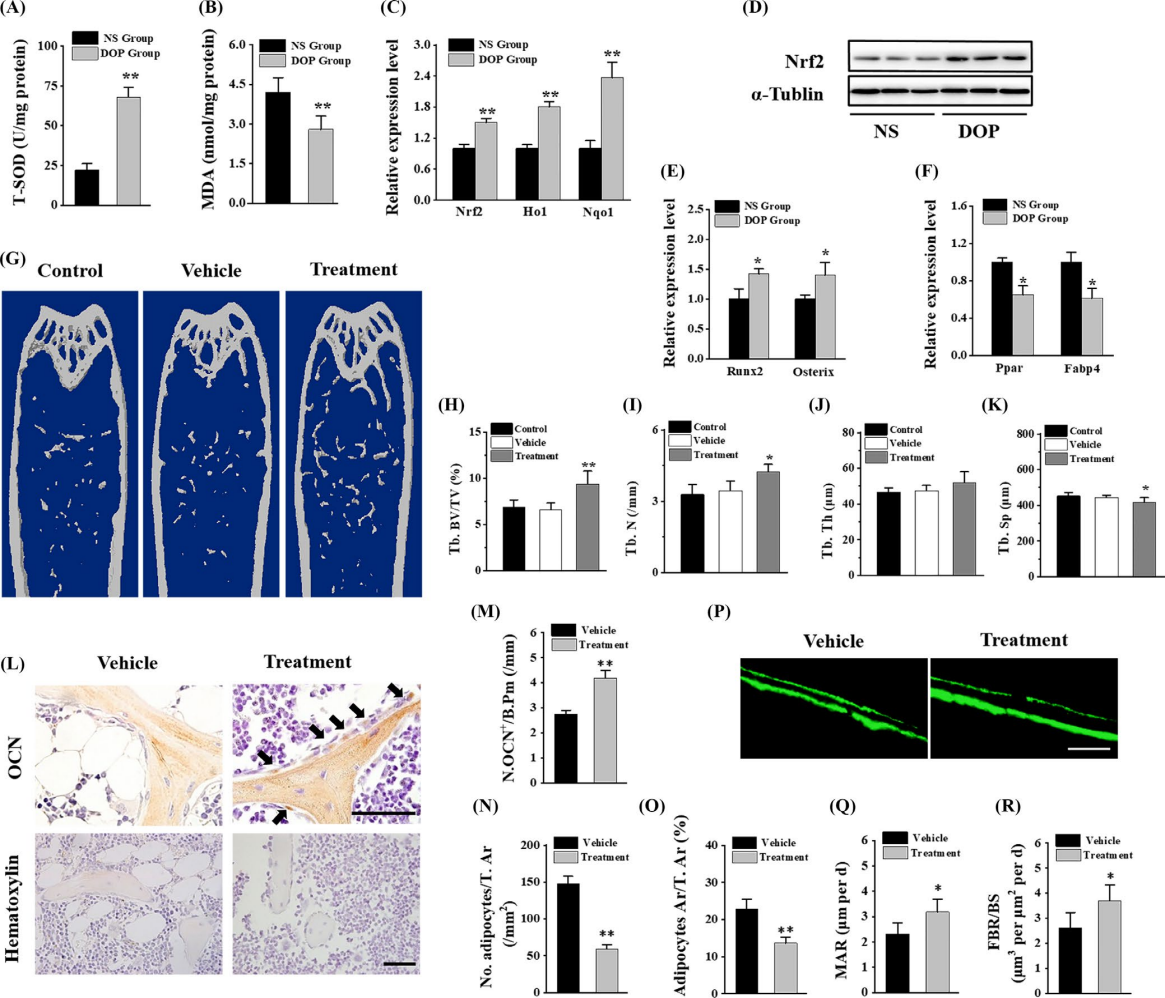
Mice treated with DOP present decreased oxidative stress and increased bone formation. Twelve‐month‐old mice were orally administrated with DOP at dosage of 150 mg/kg every day for 3 mo. (A) SOD level in BMSCs. (B) MDA level in BMSCs. (C) qRT‐PCR analysis of the levels of Nrf2, Ho1, Nqo1 expression in BMSCs. (D) Western blot analysis of the levels of Nrf2 expression in BMSCs. (E) qRT‐PCR analysis of the relative levels of Runx2 and Osterix in BMSCs. (F) qRT‐PCR analysis of the relative levels of Pparγ and Fabp4. (G‐K) Representative microcomputed tomography (μCT) images (G) and quantitative μCT analysis of trabecular (H‐K) in femora from DOP‐treated mice and their control group. (L‐O) Representative images of osteocalcin staining (L, top) and haematoxylin staining (L, bottom) and quantification of the number of osteoblasts (M) and the number and area of adipocytes (N and O) in distal femora. Scale bar: 50 μm. (P‐R) Representative images of calcein double labelling of trabecular bone (P) with quantification of mineral apposition rate (MAR) (Q) and bone formation rates (BFRs) per bone surface (BFR/BS) (R). (Scale bar: 25 µm. N = 6 mice in each group from three independent experiments). Data are represented as mean ± SD of three individual experiments Statistical significance was determined using Student's *t* test for A‐C, E‐F and M‐R, and analysis of variance (ANOVA) for H‐K. *P < 0.05. **P < 0.01

To investigate the therapeutic effects of DOP in oxidative stress and age‐related osteoporosis in vivo, we treated the 15‐month‐old mice with normal saline (NS) and DOP, respectively. DOP was orally administrated with the dose of 150 mg/kg once daily for 3 months. Notably, DOP significantly decreased the oxidative stress damage (Figure 5A,B) and increased Nrf2 expression in vivo (Figure 5C,D). qRT‐PCR analysis suggested a shift from adipogenetic differentiation to osteoblastic differentiation in BMSCs (Figure 5E,F). Microcomputed tomography (μCT) revealed that mice treated with DOP had significantly higher trabecular bone volume, number and lower trabecular separation than the control group (Figure 5G‐K). Moreover, histological and immunohistochemical analyses of femora showed decreased number and area of adipocytes in bone marrow and increased number of osteoblasts on the trabecular bone surfaces in DOP‐treated mice compared with control group (Figure 5L‐O). Calcein double labelling confirmed that DOP‐treated mice had higher bone formation rates (BFRs) and mineral apposition rate (MAR) than control group (Figure 5P‐R). Taken together, these results show that DOP could prevent bone loss and MAT accumulation by attenuating oxidative stress in aged mice.

## 4 DISCUSSION

Age‐related bone loss is becoming a growing public health problem as the progressive ageing population.^1^, ^2^, ^3^ It urgently requires us to find some potential therapeutic targets and agents to prevent the development of bone loss.^29^, ^30^, ^31^ In this study, we confirmed the potential therapeutic target of NRF2 in age‐related bone loss and the protective role of DOP in attenuating the oxidative stress–impaired osteoblast differentiation. It is well established that oxidative stress is prerequisites for the progression in skeletal ageing and osteoporosis.^3^, ^5^, ^32^ Extensive researches suggested that oxidative stress accumulation was responsible for lineage commitment of BMSCs and the bone‐fat imbalance. Yu et al recently showed PGC‐1a, a master regulator of oxidative metabolism, controlled skeletal stem cell fate and bone‐fat balance in osteoporosis and skeletal ageing.^14^ In accordance with previous studies, our results showed a higher level of oxidative stress and lower level of antioxidant activity in aged group compared to young group. Furthermore, it was clear that H_2_O_2_‐induced oxidative stress promoted adipogenesis of BMSCs at the expense of osteogenesis, which could be reversed by antioxidant activity of DOP. The elevated antioxidative enzymes in BMSCs cultured with the ADM or ODM may be caused by the response of H_2_O_2 _stimulation. Recent study identified that the deficiency of NRF2 antioxidant pathway was a driver mechanism in premature ageing disorder Hutchinson‐Gilford progeria syndrome (HGPS), and the impairment of NRF2 transcriptional activity would cause oxidative stress and related progeroid phenotypes including bone loss.^33^ These results indicated that NRF2 antioxidant activity was dispensable for age‐related diseases.

NRF2 is regarded as the master regulator of the cellular antioxidant response and a therapeutic target of oxidative‐mediated diseases.^34^, ^35^, ^36^ It is also a controversial player in bone metabolism.^28^, ^37^, ^38^, ^39^ Preliminary reports suggested overexpression of Nrf2 enhanced the potential of BMSCs to differentiate into osteogenic lineage and inhibited the potential to differentiate into adipogenic lineage.^34^, ^38^ Moreover, the deletion of Nrf2 in mice leads to a lower bone mass and an bone microarchitecture.^40^, ^41^ In our study, we utilized hBMSCs from different age subjects to detect the relationships among age‐related cell fate, NRF2 gene expression and oxidative stress. Interestingly, we found that the level of NRF2 was decreased with ageing in BMSCs, accompanied by increasing oxidative stress and shift of cell fate in BMSCs. It indicated Nrf2 was a key factor in BMSCs lineage commitment. However, some studies suggested that Nrf2 was a detrimental factor in bone haemostasis.^37^, ^40^, ^42^ We speculated that the previous reported inconsistent effects were associated with some confounding factors, such as different sex, age, genetic background and even cross communications between cells in skeletal system or communication with other organs in the global Nrf2 knockout mice.^37^, ^43^

DOP is a major ingredient in Dendrobium officinale and known with its antioxidant activity.^44^ Some work suggested the protective role of Dendrobium officinale in bone mass and natural ageing‐induced premature ovarian failure.^45^ In consistent with previous protective results, our study found that DOP remarkably increased osteogenesis and decreased adipogenesis in BMSCs, which could contribute to prevent age‐related bone loss. Moreover, some studies showed that DOP had a positive effect on activating Nrf2 signalling.^27^ In this paper, we demonstrated that BMSCs showed deficient osteogenesis and enhanced adipogenesis when exposure to oxidative stress, and the treatment of DOP could reverse this effect. However, this effect rescued by DOP treatment was absent in Nrf2‐knock‐down BMSCs. Moreover, we also validated its anti‐osteoporosis activity assessed by remarkably restored bone loss and MAT accumulation in the treated mice, which were accompanied with upregulated defence against oxidative stress.

**Figure 6:**
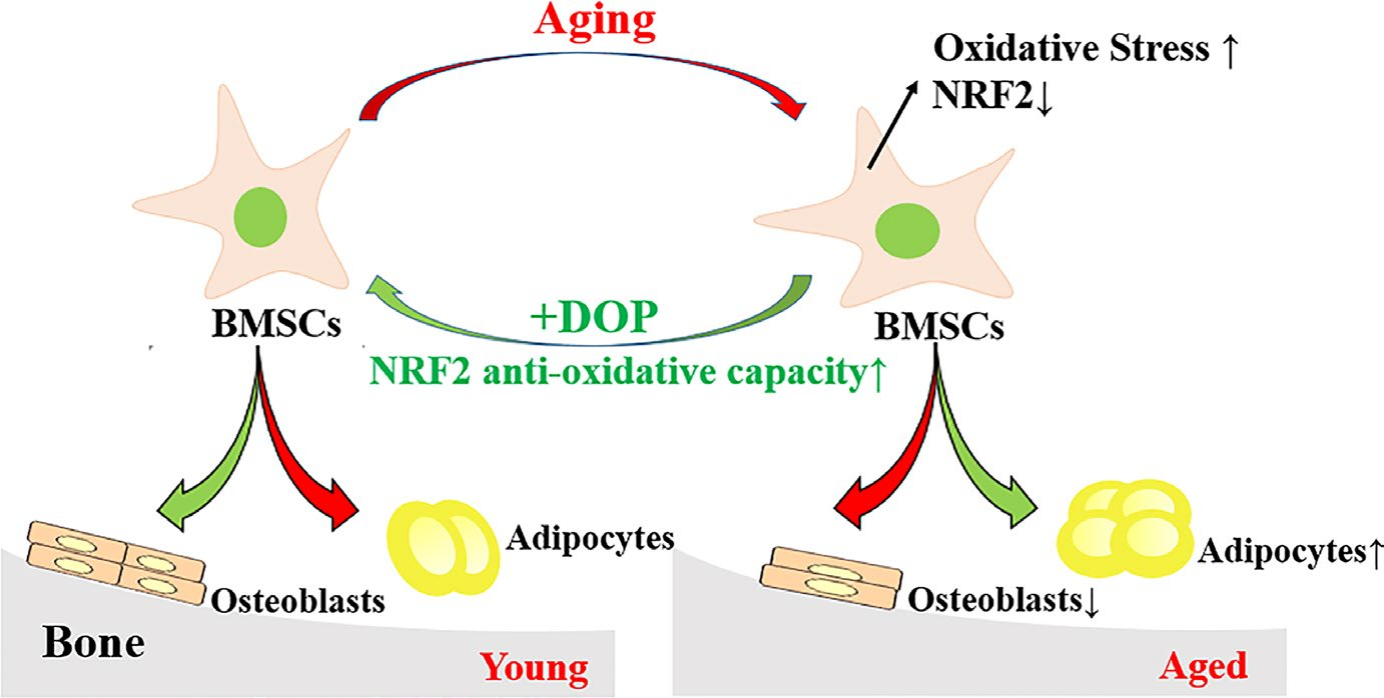
Schematic illustration of DOP‐regulating age‐related osteogenesis and adipogenesis in bone marrow via activating NRF2 signalling. DOP has a positive effect on restoring the ageing‐induced oxidative stress and imbalanced fate shift between adipocytes and osteoblasts in BMSCs through the antioxidant NRF2 signalling

Together, these findings imply that DOP controls age‐related lineage commitment of BMSCs and bone‐fat balance by activating antioxidant NRF2 signalling (Figure 6). Our study demonstrates a rationale therapeutic target and a potential anabolic agent in age‐related bone loss.

## CONFLICT OF INTERESTS

The authors have declared that no conflict of interest exists.
