# Supplementary material for: Dendrobium officinale polysaccharides regulate age‐related lineage commitment between osteogenic and adipogenic differentiation
Source: Cell Prolif. 2019 Apr 30;52(4):e12624. doi: 10.1111/cpr.12624 (PMC6668967; doi:10.1111/cpr.12624)
Supplement: Supplementary file 1 [file CPR-52-e12624-s001.docx]

**Figures**

**Supplementary Figure 1. Effects of melatonin on cell viability.** MSCs were seeded in 96-well plates at a density of 8 × 103 cells/well and then treated with indicated concentrations of DOP for 2 days. Cell viability was determined using the MTT assay. Data are represented as mean ± SEM of three individual experiments (n = 3). Statistical significance was determined using Student's.

**Tables**

**Supplementary Table 1. Primers of mice for quantitative RT-PCR**

| Gene | Primers |
| --- | --- |
| Actin-F | GGCTGTATTCCCCTCCATCG |
| Actin-R | CCAGTTGGTAACAATGCCATGT |
| Pparg- F | ATGGTT GACACAGAGATGC |
| Pparg-R | GAATGCGAGTGGTCTTCC |
| Fabp4-F | AAGGTGAAGAGCATCATAACCCT |
| Fabp4-R | TCACGCCTTTCATAACACATTCC |
| Nrf-2-F | TCCGCTGCCATCAGTCAGTC |
| Nrf-2-R | ATTGTGCCTTCAGCGTGCTTC |
| Nqo-1-F | CAAGTTTGGCCTCTCTGTGG |
| Nqo-1-R | AAGCTGCGTCTAACTATATGT |
| Ho-1-F | AACAAGCAGAACCCAGTCTATGC |
| Ho-1-R | AGGTAGCGGGTATATGCGTGGGCC |

**Supplementary Table 2. Primers of human for quantitative RT-PCR**

| Gene | Primers |
| --- | --- |
| ACTIN-F | CGTGGACATCCGCAAAGA |
| ACTIN-R | TCGTCATACTCCTGCTTGCTG |
| NRF2-F | TCAGCGACGGAAAGAGTATGA |
| NRF2-R | CCACTGGTTTCTGACTGGATGT |
| NQO1-F | GAAGAGCACTGATCGTACTGGC |
| NQO1-R | GGATACTGAAAGTTCGCAGGG |
| HO-1-F | ATGCCCCAGGATTTGTCAGA |
| HO-1-R | AAGTAGACAGGGGCGAAGAC |
